# Heterostrain and temperature-tuned twist between graphene/*h*-BN bilayers

**DOI:** 10.1038/s41598-023-31233-3

**Published:** 2023-03-16

**Authors:** Xing Yang, Bin Zhang

**Affiliations:** State Key Laboratory of Mechanics and Control of Mechanical Structures, and College of Aerospace Engineering, Nanjing University of Aeronautics and Astronautics, Nanjing, 210016 China

**Keywords:** Graphene, Structural properties, Two-dimensional materials

## Abstract

Two-dimensional materials stacked atomically at small twist angles enable the modification of electronic states, motivating twistronics. Here, we demonstrate that heterostrain can rotate the graphene flake on monolayer *h*-BN within a few degrees (− 4° to 4°), and the twist angle stabilizes at specific values with applied constant strains, while the temperature effect is negligible in 100–900 K. The band gaps of bilayers can be modulated from ~ 0 to 37 meV at proper heterostrain and twist angles. Further analysis shows that the heterostrain modulates the interlayer energy landscape by regulating Moiré pattern evolution. The energy variation is correlated with the dynamic instability of different stacking modes of bilayers, and arises from the fluctuation of interlayer repulsive interaction associated with *p*-orbit electrons. Our results provide a mechanical strategy to manipulate twist angles of graphene/*h*-BN bilayers, and may facilitate the design of rotatable electronic nanodevices.

## Introduction

Two-dimensional (2D) materials become an ideal platform for studying fundamental physical properties of atomic-scale systems and promise in massive engineering applications^1^. Over the past years, various 2D material interfaces (e.g., graphene/graphene, graphene/*h*-BN, and graphene/MoS_2_) have been synthesized by emerging nanotechnology^2^. In 2D materials systems, the interlayer twist angle (*θ*) between adjacent layers provides a variety of electronic phases, triggering exotic phenomena such as unconventional superconductivity, nonlinear optics, structural superlubricity, as well as thermal anisotropy^3–5^. The desired physical properties are sensitive to the Moiré superlattice, which requires precise control of *θ*.

Mechanical stacking and chemical synthesis techniques have been developed to fabricate twisted 2D materials^6–11^. For example, selected area electron diffraction analysis validated the presence of twisted regimes in MoS_2_/*h*-BN heterostructures grown by chemical vapour deposition^6^; Raman spectra characterization confirmed a series of graphene/graphene samples with *θ* = 0º–30º obtained through mechanical transfer^10^. The stability of twisted samples is dominated by van der Waals forces which provide resistance to interlayer relative motion (e.g., sliding and rotating)^12^. However, *θ* cannot be modified again once bilayers assembled, and the dynamic control of *θ* is still lacking.

Experiments observed interlayer dynamic twist at superlubric interfaces (a state of ultra-low friction). For instance, misaligned graphene/*h*-BN interfaces experienced spontaneous rotation during annealing^13,14^. This phenomenon was also found in graphene/graphene systems at lower temperatures^15,16^. From an energy point of view, incommensurate contacts (*θ* > 0°) have flatter potential energy landscapes due to lattice mismatch-induced cancellation of interlayer interactions, thereby reducing the rotational resistance^17,18^. Once interfaces approach commensurate contacts (*θ* = 0°), significant potential energy corrugation appears. Numerous atoms are trapped in the local minima of potential energy landscapes, e.g., the center of mass (COM) of each hexagonal lattice^19^, leading to elevated rotational resistance, which are further related to the *sp*^2^-hybridized electron clouds, as demonstrated in previous theoretical calculations^20,21^. Therefore, the regulation of interlayer interactions should be a viable way to modulate twist.

In this work, we realize the dynamic twisting of the graphene flake on monolayer *h*-BN by modulating heterostrain and temperature. Using a validated bilayer model, we uncover an energy mechanism that dominates the twisting path, which is rationalized by a combined analysis of the interlayer energy landscape, atomic stacking modes, and electronic energy bands. The results are expected to help develop rotatable micro-electro-mechanical devices.

## Model, results and discussion

**Figure 1:**
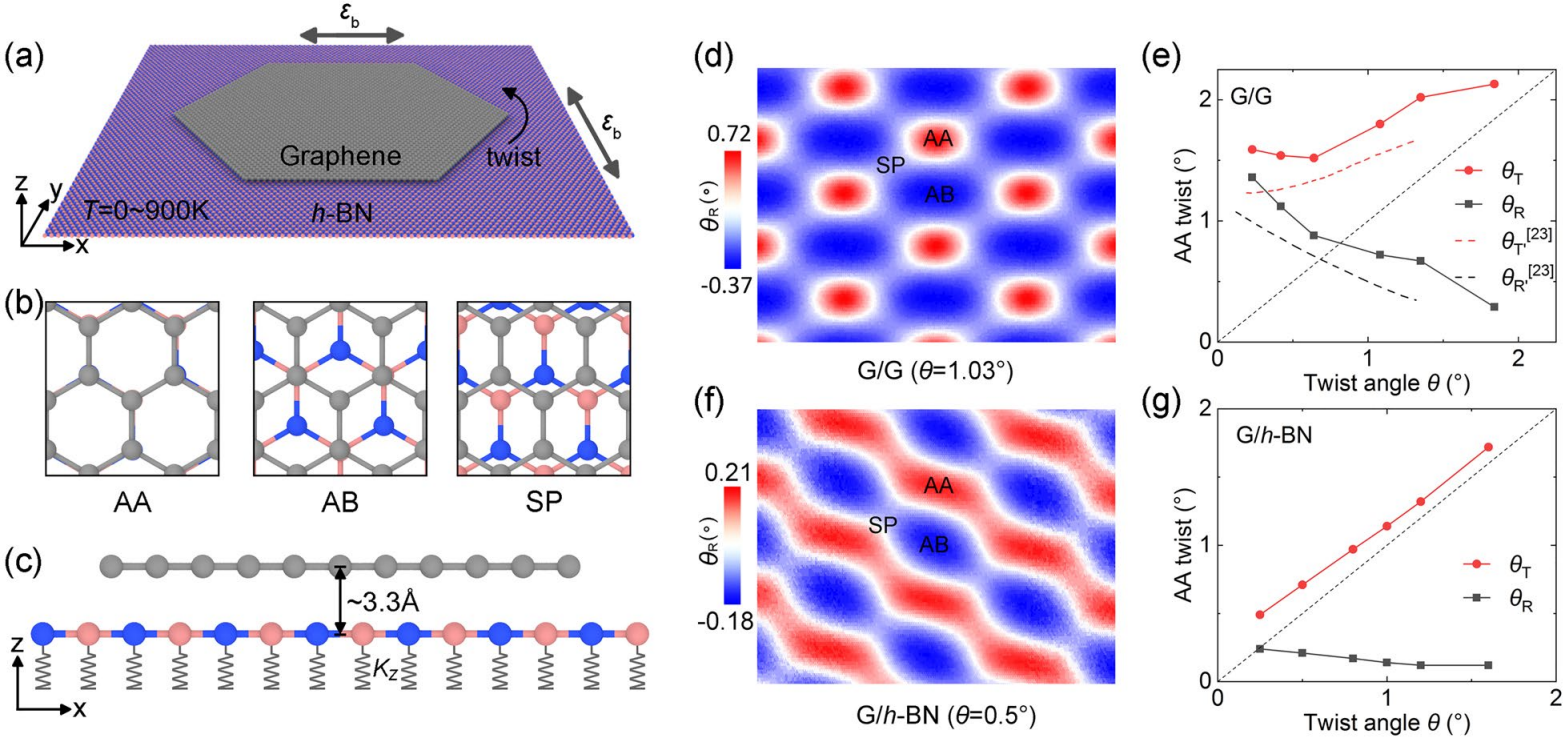
Schematic of the G/*h*-BN bilayer model and its verification. (**a**) A hexagonal graphene flake with width of 13.7 nm is placed on an infinite *h*-BN monolayer under heterostrain $\varepsilon_{{\text{b}}}$ (0–10%) and temperature *T* (100–900 K). (**b**) Atomic configuration of three initial stacking modes, AA, AB, and SP. (**c**) The lower *h*-BN layer is supported by independent linear springs with stiffness *k*_z_ = 2.7 nN/nm. (**d**–**g**) Validation of the bilayer models. (**d**) The distribution of local twist angles *θ*_R_ is consistent with the previous experiments^23^. (**e**) The local AA twist with the applied twist angle agrees with the experiments^23^. (**f**,**g**) Results of G/*h*-BN are analogous to those of G/G.

Molecular dynamics (MD) methods are used to study the interlayer rotation of the graphene/*h*-BN (G/*h*-BN) bilayer. Figure 1 demonstrates the MD model. A hexagonal graphene flake with width of 13.7 nm is placed on the infinite *h*-BN monolayer (Fig. 1a). Three stacking modes are considered (Fig. 1b). Independent linear springs are used to support the substrate *h*-BN layer (Fig. 1c)^22^. Then, twisted initial configurations are built by rotating the flake around its COM to *θ* = 30º. Additionally, the in-plane COM of the graphene flake is constrained to facilitate quantitatively study of the twist. The simulation setup is detailed in “Methods”.

To validate the model, we conduct structural relaxations for both G/G and G/*h*-BN bilayers at temperature *T* = 1 K, and calculate the local twist angle *θ*_R_ of the upper graphene. For each hexagonal lattice in graphene, *θ*_R_ is defined as the orientation difference before (*θ*_unrelax_) and after (*θ*_relax_) relaxations, i.e., *θ*_R_ = *θ*_relax_ − *θ*_unrelax_. For G/G with *θ* = 1.03° (Fig. 1d), *θ*_R_ exhibits distribution characterized by Moiré patterns, which is consistent with recent experimental studies^23^. We note that the Moiré patterns do not exhibit triple symmetry, which is related to the local lattice distortions, out-of-plane fluctuations, and interlayer sliding. Effects of *θ* on the rotational reconstruction are shown in Fig. 1e, where *θ*_T_ = *θ*_R_ + *θ* is the interlayer fixed-body twist. The curves also agree with the experiments (dashed lines)^23^. For G/*h*-BN with *θ* = 0.5°, the local twist displays periodic fluctuations (Fig. 1f) and a weaker trend than G/G (Fig. 1g). These comparisons demonstrate that our model can reasonably describe the nano-stacking of G/*h*-BN.

**Figure 2:**
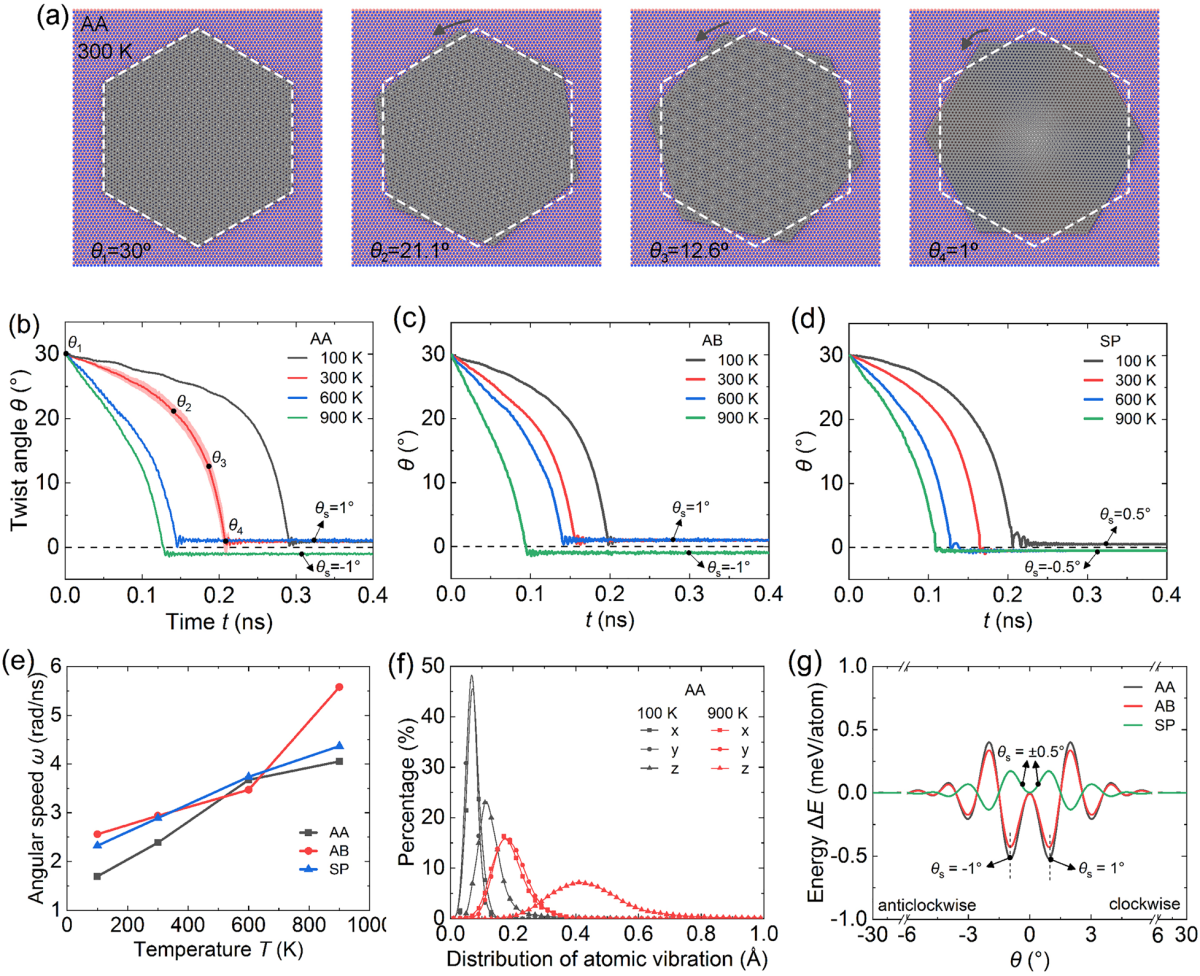
Temperature-modulated dynamic twisting of the graphene flake on monolayer *h*-BN. (**a**) Typical twist at 300 K. (**b**–**d**) Twist angles *θ* vary with time for AA, AB, and SP modes at series temperature. Each curve has only slight thermal fluctuations, as demonstrated by the red error band for AA at 300 K. (**e**) Average angular speed $\omega$ as a function of temperature. $\omega$ is calculated as the *θ* difference from the initial value to the first local minimum divided by the time. (**f**) The elevated temperature (100 to 900 K) mainly enhances the out-of-plane vibration (0.1 to 0.4 Å) of atoms in the flake. Each data point represents an interval width of 0.02 Å. (**g**) Interlayer energy versus *θ* at 0 K. *θ*_s_ in (**b**–**d**) approaches the local minima of curves in (**g**).

The evolution of interlayer twist at a series of *T* = 100–900 K is studied in Fig. 2. As simulation develops, Moiré patterns form and stabilize as *θ* approaches 1°, see Fig. 2a. A quantitative assessment of the dynamic process is conducted by tracking instantaneous *θ*. For the AA mode at *T* = 100 K (Fig. 2b), the flake twists slowly at the beginning with a slight drop of *θ*. Then the speed accelerates, and the flake finally stops rotation at a stable angle of *θ*_s_ = 1°. At higher *T*, we observe increasingly fast speeds, i.e., *θ*_s_ =  − 1° at *T* = 900 K. The other two cases AB and SP (Fig. 2c,d) are analogous to AA. Both of them exhibit spontaneous twists and stabilize at specific *θ*_s_. Differently, the SP mode produces *θ*_s_ = 0.5° at *T* = 100 K and -0.5° at *T* ≥ 300 K.

The average angular speed is calculated as *ω* = Δ*θ*/Δ*t*. Δ*θ* is the angular change from the beginning to the stable stage, and Δ*t* is the elapsed time. In Fig. 2e, *ω* increases by about two times at the temperature range 100–900 K, i.e., 1.7 to 4.1 rad/ns for AA, 2.6 to 5.6 rad/ns for AB, and 2.3 to 4.4 rad/ns for SP. These velocities are around several 10^9^ rad/s that can create nanomechanical systems with gigahertz modulating frequency^24^. We also consider the cases without in-plane constraint of COM of the graphene flake, and the average angular speed does not show apparent dependence on the in-plane constraint of COM despite MD fluctuations (see Supplementary Figs. S1 and S2). In addition, the rising *ω* also implies a decrease in the interlayer friction, consistent with the thermally activated PT model^25^. Further analysis shows that the increasing temperature mainly enhances the out-of-plane vibration (0.1 Å to 0.4 Å) of atoms in the flake, while the in-plane x- and y-direction undergo mild variation less than 0.1 Å (Fig. 2f). This reinforcement of out-of-plane vibration makes it easier for atoms to overcome friction, resulting in twisting acceleration.

However, the rotation in all cases finally stopped without exception, indicating that the friction cannot be easily overcome by tuning temperature only. In Fig. 2g, we check the interlayer potential energy at 0 K (to eliminate the thermal noise). The fluctuating curves reveal multiple local minima at specific *θ*, which constitutes a series of energy barriers to self-rotation. For AA and AB, the most significant energy barriers are at *θ* =  ± 0.9° and ± 3°, despite the slight peak differences. Whereas for SP, they are concentrated at *θ* = 0° and ± 2°. Apparently, the distribution of energy barriers depends on the stacking mode, which explains the diverse *θ*_s_ in our simulations. We note that some *θ*_s_ are not located strictly at the saddle points of curves because the lattice deformation at finite temperatures slightly alters the energy landscape^26,27^. Briefly, the twist always follows the direction of the local minimum of potential energy. If the interlayer energy landscape can be dynamically modulated, a continuous twist could be achieved.

**Figure 3:**
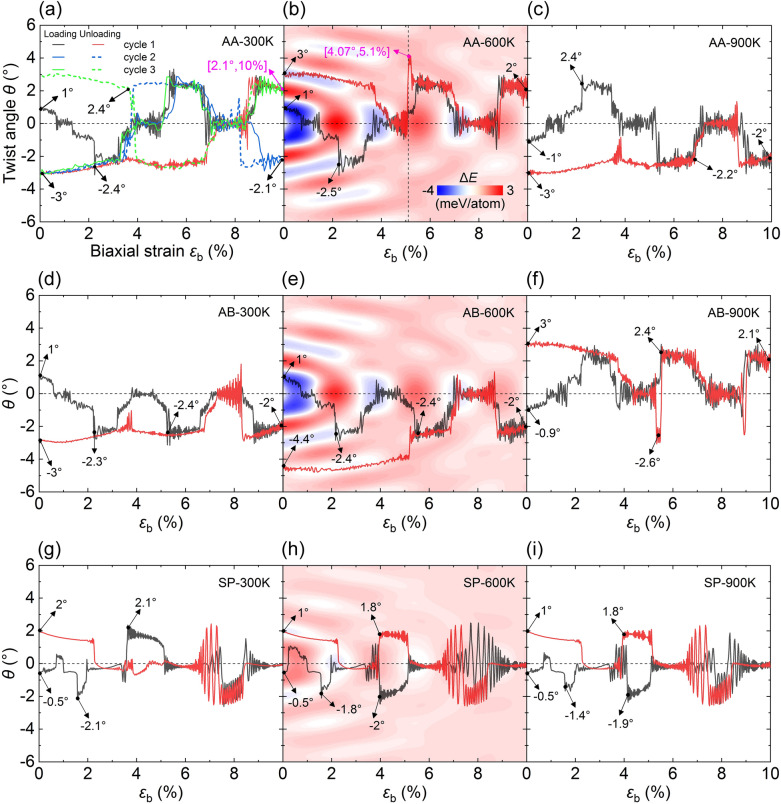
Heterostrain (strain rate = 0.025/ns) and temperature-modulated dynamic twisting of the graphene flake. (**a**–**c**) for the AA mode, (**d**–**f**) the AB mode, and (**g**–**i**) the SP mode. The first, second, and third cycle strain loading are presented in (a), and only the first cycle is plotted in (**b**–**i**). Interlayer energy landscapes are superimposed in (**b**), (**e**), and (**h**) to illustrate the interlayer energy-dominated twisting path. Each data is calculated using the energy value of *θ* = 0° as the benchmark. The legend in (**b**) also applies to (**e**) and (**h**).

Heterostrain, the in-plane deformation of one layer relative to another, can directly modulate the interlayer interaction by manipulating the ratio of lattice constants^28,29^. We consider the biaxial strain of *h*-BN by gradually loading and unloading at a constant strain rate of 0.025/ns. In Fig. 3, each curve starts with a corresponding *θ*_s_. For AA (Fig. 3a), the flake twists anticlockwise from *θ* = 1° to -2.4° at $\varepsilon_{{\text{b}}}$ = 0–2.2%. Then, *θ* fluctuates around − 2° from 2.2 to 3.2%. As strain increases further, *θ* attains the positive maximum 2.4° at $\varepsilon_{{\text{b}}}$ = 5.2%. After that, *θ* alternates between 0° and 2.4° at elevated strain, and then gradually varies from 2.1° to − 3° in the unloading stage (see Supplementary video). The dynamic twisting is repeatable considering the second and third cyclic loads, see Fig. 3a. At higher temperatures (Fig. 3b,c), the angular ranges are close to the above, i.e., − 2.5° to 4.1° for *T* = 600 K and − 3° to 2.4° for *T* = 900 K. Results of AB are shown in Fig. 3d–f. *θ* varies between − 3° to 1°, − 4.4° to 1°, and − 2.6° to 3° for *T* = 300, 600, and 900 K, respectively. In comparison, the SP mode depicts a slightly smaller amplitude between − 2° and 2° (Fig. 3g–i). Therefore, heterostrain combined with temperature can realize the interlayer twist. The stepped curves further illustrate that *θ* can be stabilized at specific values when a proper strain is applied (e.g., $\varepsilon_{{\text{b}}}$ = 4.0–6.7% for *θ* = 2.4° in the AA mode)^30^.

To deepen the understanding of twist, we analyze the interlayer energy landscapes (0 K) by calculating the interlayer energy increment (Δ*E*) relative to *θ* = 0° under evaluated $\varepsilon_{{\text{b}}}$ and *θ*. In Fig. 3b, blue and red regions represent the local low-energy and high-energy domains, respectively. The heterostrain modulates the interlayer energy, making the original position unstable and thus the flake rotates. Taking AA mode as examples, the potential energy of *θ* = 0° alters from a local maximum to a minimum when $\varepsilon_{{\text{b}}}$ increases from 0 to 1%, and then attains a local maximum again at $\varepsilon_{{\text{b}}}$ = 2%. This alternation is also observed in Fig. 3e,h. The curve inflection points always occur in the region with significant energy change, suggesting that the twist tends to proceed along the direction of potential energy gradients for all cases. Considering that the strain rate in usual experiments is far lower than that in our atomistic simulation, a smoother angular modulation would be obtained since the longer relaxation time enables the flake to twist toward the optimal path.

Why does the interlayer potential energy vary with twist? Previous studies attribute the energy variation to Moiré pattern evolution. Within a complete Moiré superlattice, the equivalent energy barrier approaches zero^31^. When the center of Moiré patterns cuts through the flake edge, the interlayer energy attains a local extreme value^32^. Although these studies have provided qualitative insight into the interlayer interaction of layered 2D materials, it still lacks further explorations.

**Figure 4:**
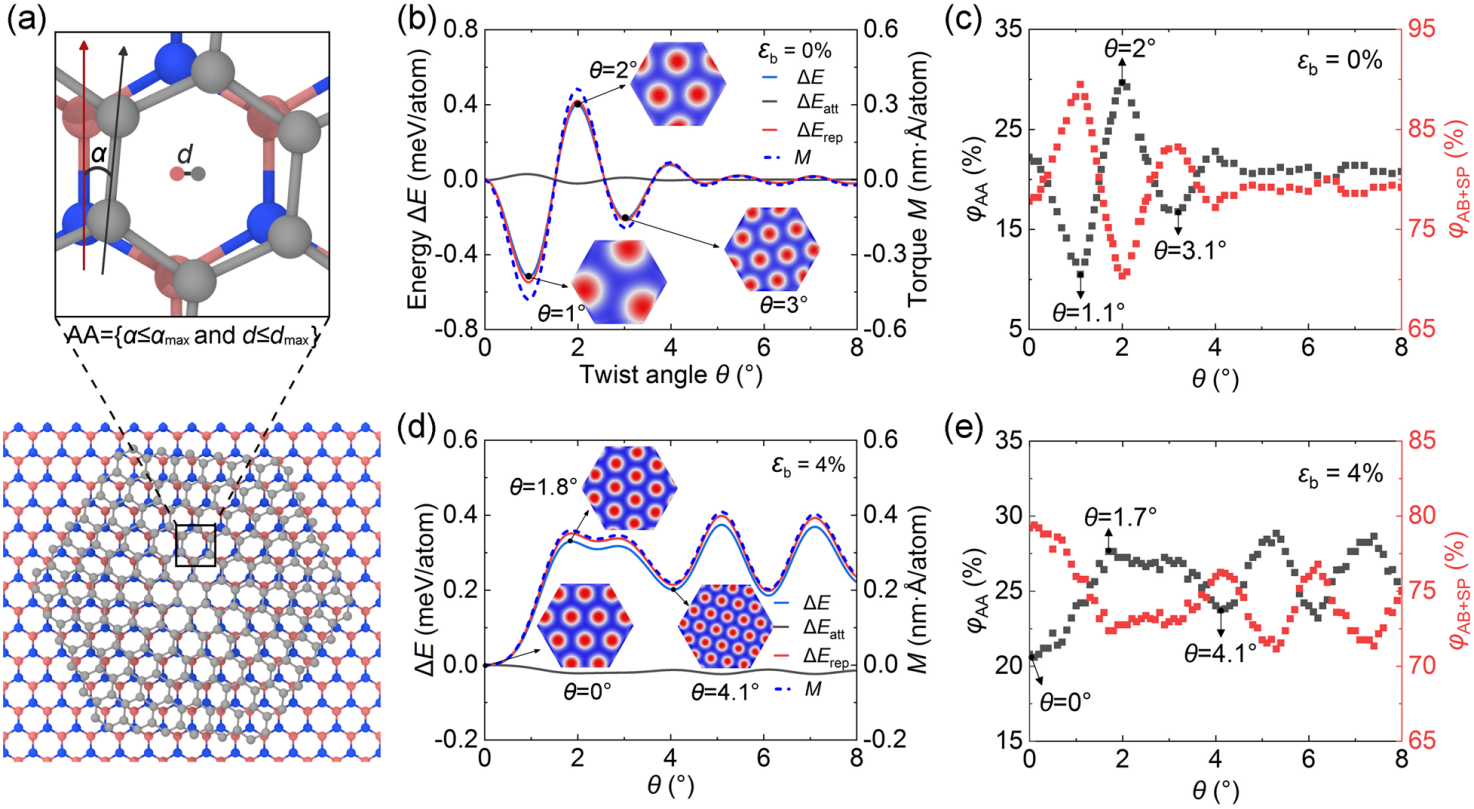
Correlation between stacking modes and interlayer energy. (**a**) Schematic of the AA mode. *d* represents the distance between the centroids of two lattices, and *α* denotes the orientation difference. When *d* < *d*_max_ and *α* < *α*_max_, the interface lattice is considered to be in the AA mode. Here, *d*_max_ = 0.5 Å and *α*_max_ = 8°. By calculating the lattice one by one, we can get the proportion of the AA mode in the entire flake. (**b**,**d**) Variation of the interlayer energy (Δ*E*) modulated by heterostrain. Δ*E* has been decomposed into the attractive (Δ*E*_att_) and repulsive (Δ*E*_rep_) components. The rotational torque is calculated by *M* = $\partial{E}$/$\partial{\theta}$. Insets show the evolution of the AA (red) and AB + SP (blue) modes. (**c**,**e**) Ratios of different stacking modes versus *θ*. *φ*_AA_ and *φ*_AB+SP_ represent the ratio of AA and AB + SP stacking modes, respectively. Arrows indicate local maxima and minima.

Moiré pattern evolution involves the alternation of AB, SP, and AA stacking modes. Among them, the AA mode is the least stable (highest energy), SP mode the metastable (medium energy), and AB mode the most stable (lowest energy). Insets in Fig. 4b exhibit several distributions of the AA (red) and AB + SP (blue) modes. The AB + SP mode dominates at small *θ*. With the flake twist, all modes gradually shrink, forming more superlattice arrays. This implies that the fluctuation of interlayer energy should be related to the dynamic evolution of these modes. An algorithm based on the lattice displacement vector is developed to determine their quantities. As depicted in Fig. 4a, for any pair of heterogeneous hexagonal lattices, when the COM distance *d* and the lattice orientation difference *α* satisfy *d* ≤ *d*_max_ and *α* ≤ *α*_max_, we distinguish the graphene lattice as the AA mode. By examining all the lattices one by one, we obtain the ratio of the AA mode relative to all graphene lattices (*φ*_AA_). Naturally, the rest is the ratio of the AB + SP mode (*φ*_AB+SP_). Results of *d*_max_ = 0.5 Å and *α*_max_ = 8° are given in Fig. 4c. Surprisingly, *φ*_AA_ mirrors the same trend with the Δ*E* curve, while *φ*_AB+SP_ is the opposite. For example, the local minima of *φ*_AA_ at *θ* = 1.1° and 3.1°, as well as the local maximum at *θ* = 2°, approach the energy extreme points at *θ* = 1°, 2°, and 3°, respectively. Once *φ*_AA_ (or *φ*_AB+SP_) flattens out at larger *θ* (e.g., *θ* > 5°), the energy hardly fluctuates. We have confirmed that different combinations of *d*_max_ and *α*_max_ only cause the amplitude fluctuation of *φ*_AA_ and *φ*_AB+SP_ but do not change their trends. In another case (Fig. 4d), the biaxial stretching modifies the Moiré pattern evolution. The latter is described by the Moiré wavelength formula $L = pa_{{\text{G}}} /\sqrt {1 + p^{2} - 2p\cos (\theta )}$ and $p = a_{{{\text{BN}}}} (1 + \varepsilon_{{\text{b}}} )/a_{{\text{G}}}$, where $a_{{\text{G}}}$ and $a_{{{\text{BN}}}}$ are the lattice constants of graphene and *h*-BN, respectively^33^. Nevertheless, *φ*_AA_ (*φ*_AB+SP_) still follows (goes against) the trend of energy curves (Fig. 4e). Accordingly, interlayer energy fluctuations (or energy barriers) can be correlated with the dynamic instability of different stacking quantities, which eventually leads to the change of rotational resistance (see torque *M* curves in Fig. 4b,d).

Generally, the interlayer energy in G/*h*-BN arises from the long-range attractive energy (*E*_att_) and the short-range repulsive energy (*E*_rep_). The former is dominated by inter-atomic dispersive interactions that accounts for the adhesion force of adjacent layers, and the latter reflects the Pauli repulsion^34^. Δ*E* can be decomposed into Δ*E* = Δ*E*_att_ + Δ*E*_rep_. In Fig. 4b, Δ*E*_rep_ almost overlaps with Δ*E*, while Δ*E*_att_ is relatively insensitive to *θ* (~ 0.03 meV/atom). This means that the interlayer energy fluctuation is primarily contributed by the short-range repulsive interaction, which is further examined in Fig. 4d. Similar results have been obtained in previous studies. For example, using quantum-chemistry-based MD methods, Onodera et al.^35^ found that the predominant Coulomb repulsion interactions between two sulfur layers in different MoS_2_ directly affect its lubrication properties. The short-range repulsion is essentially from electron interaction. In our model systems, atoms of graphene and *h*-BN are bonded together in *sp*^2^ hybridization. For each atom, one *s* orbit and two *p* orbits form three co-planar σ bonds within monolayer, and the remaining *p* orbit forms the interlayer π-bond^36^. This creates a closed π electron cloud system perpendicular to the lattice plane, which leads to the overlap of π electron clouds and the overall repulsive force^37^.

**Figure 5:**
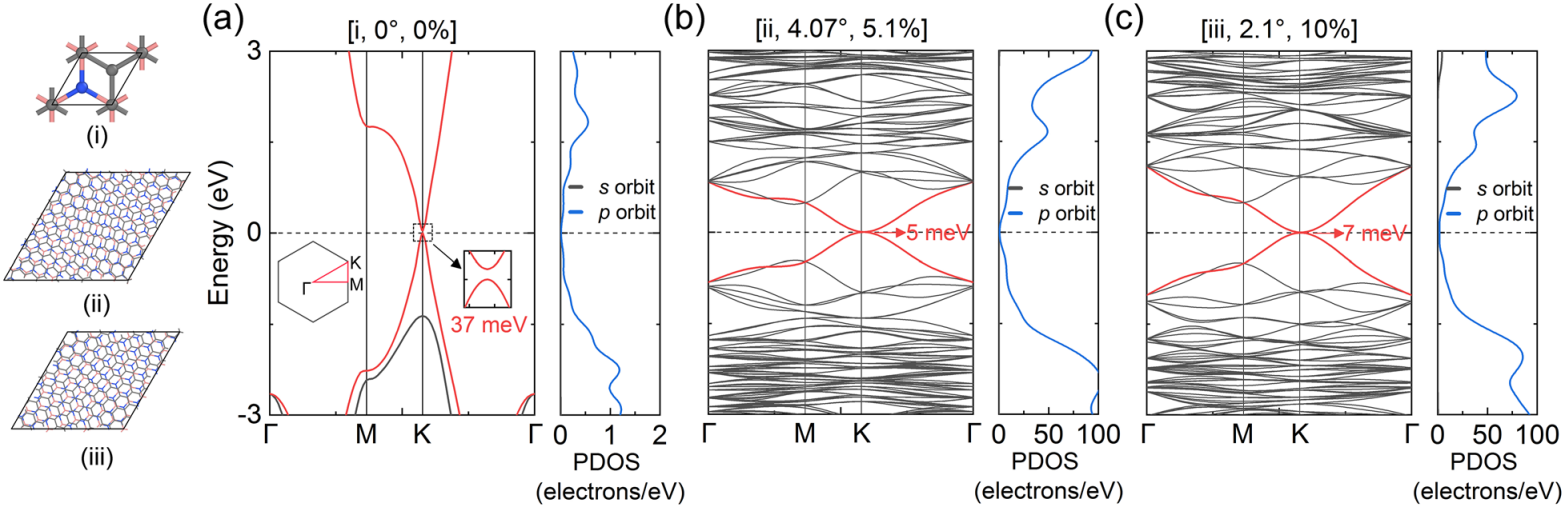
Heterostrain-modulated energy bands and partial densities of state (PDOS). Left panel: (i), (ii), and (iii) present the bilayer supercells of $[\theta = 0^\circ ,\varepsilon_{{\text{b}}} = 0\% ]$, [4.07°, 5.1%], and [2.1°, 10%] in Fig. 3a,b (see magenta arrows) with 10, 362, and 284 atoms in the supercells, respectively. Corresponding results are shown in (**a**–**c**). Inset in (**a**) outlines the integration path in the first Brillouin zone. Horizontal dotted lines indicate the Fermi level. Red curves mark the valence band maximum and the conduction band minimum, which determine the band gap. In each PDOS, the contribution of *s* orbits is too small to be seen.

The interactions of electron clouds might be also modulated by heterostrain and twist angles. To illustrate this, we calculate the electronic structures of twisted bilayers (see “Methods” for detailed setup). It should be noted that the results presented here are for G/*h*-BN supercells rather than the hexagonal graphene flake itself to qualitatively analyze the effect of heterostrain on electronic structures. First, we consider *θ* = 0° at $\varepsilon_{{\text{b}}} = 0\%$ (i.e., [0°, 0%], the same elsewhere). In Fig. 5a, Energy bands (red lines) near the Fermi level (dotted lines) exhibit quadratic dispersion characteristics. A direct band gap of 37 meV is opened at the K point of the first Brillouin zone (see inset), which is slightly smaller than ~ 38 meV of Chen et al.^38–40^, but larger than ~ 17 meV of Kim et al.^41^ The partial density of state (PDOS) shows that the energy bands are mainly contributed by *p* orbits, while the *s* orbit is negligible. With the strain increasing (Fig. 5b), the band gap reduces to 5 meV at [4.07°, 5.1%], and massive flat bands appear, indicating stronger electronic localization. Furthermore, we can estimate the existence of a 0 meV band gap at specific strains and angles for the difficulty of density functional theory (DFT) calculations with giant atom amount in the supercell with small *θ*. As the strain increases further, the band gap rises slightly to 7 meV, and the energy band is still dominated by *p* orbits (Fig. 5c). Heterostrain-modulated electronic structures are also found in previous studies^42–44^. In particular, a recent experiment realized dynamically tunable heterostrain on bilayer graphene by detecting multiple absorption peaks^42^. Moreover, the symmetry of band structures was completely reconstructed by heterostrain, and there were no longer Dirac points^44^. Therefore, it is feasible to control electrical properties by regulating interlayer twist with heterostrain, which may be extended to other 2D heterostructures.

## Conclusions

The twisting graphene flake on monolayer *h*-BN under a series of temperature (100–900 K) and heterostrain (0–10%) is studied by molecular dynamics simulations and density functional theory (DFT) calculations. We find that the heterostrain of *h*-BN can twist the graphene flake within -4° to 4°, while temperature has a negligible effect. Furthermore, the twist angle stabilizes at specific values with applied constant strains, and the band gaps of bilayers can be tuned from ~ 0 to 37 meV. The heterostrain modifies the interlayer energy landscape, which makes the system incessantly reach new saddle points along the direction of energy gradients, and is correlated with the fluctuation of different stacking modes (AA, AB, and SP) along with the interaction of *p-*orbit electrons. Our strategy might provide insights for manipulating twists in 2D materials.

## Methods

### MD simulations

MD simulations are carried out with the LAMMPS package^45^. The intralayer interactions in graphene and *h*-BN are computed via the second-generation REBO and the Tersoff potential, respectively^46,47^. The interlayer interaction is described by the state-of-the-art ILP potential^48^. Prior to simulations, an energy minimization is performed utilizing the FIRE algorithm with the stopping tolerance of 10^–8^ eV/Å. Then the system is run in canonical (NVT) ensemble with Nosé-Hoover thermostat and timestep 1 fs.

### DFT calculations

The first principle calculations are performed in the framework of density functional theory. The generalized gradient approximation (GGA) of the Perdew-Burke-Ernzerhof (PBE) exchange correlation functional is employed to calculate structural optimization and electronic structures, as implemented in the CASTEP code^49^. The optimized cutoff energy for the expansion of wave functions is 500 eV. The optimized *k*-point mesh is 12 × 12 × 1 for Fig. 5a, and 3 × 3 × 1 for Fig. 5b,c. Besides, a vacuum space of 20 Å between adjacent bilayers is adopted. Structural optimization runs until the residual force ≤ 0.01 eV/Å on each atom.

## Supplementary Information

Supplementary Information 1.

Supplementary Legends.

Supplementary Video 1.

## Data Availability

All data generated or analysed during this study are included in this published article.
