# Supplementary material for: Heterostrain and temperature-tuned twist between graphene/h-BN bilayers
Source: Sci Rep. 2023 Mar 16;13:4364. doi: 10.1038/s41598-023-31233-3 (PMC10020467; doi:10.1038/s41598-023-31233-3)
Supplement: Supplementary file 1 — Supplementary Information 1. [file 41598_2023_31233_MOESM1_ESM.pdf]

Supplementary Information

Heterostrain and temperature-tuned twist between graphene/*h*-BN bilayers

Xing Yang, Bin Zhang

State Key Laboratory of Mechanics and Control of Mechanical Structures, and College of Aerospace Engineering, Nanjing University of Aeronautics and Astronautics, Nanjing 210016, China.

Corresponding author: Bin Zhang;

Tel./fax: +86 25 84891422; E-mail address: beenchang@nuaa.edu.cn

1. The configuration/constraint-dependence of the rotation of graphene flake on *h*-BN

We conduct simulations by removing the in-plane constraint of COM of the graphene flake. Each configuration is repeated four times (green curves) with different initialization parameters. The case with constraint of COM (red curves) is also added for comparison. In **Figure S1a-1c**, the flake undergoes in-plane sliding, and in **Figure S1d-1f**, the rotation time shows configuration dependence once (dark green curves). However, 3/4 green curves approach the red one in (d) and (e). In **Figure S1g**, the averaged angular speeds under unconstrained (green) and constrained (red) COM are coincide, which show that free in-plane COM has no significant effects despite the MD fluctuations.

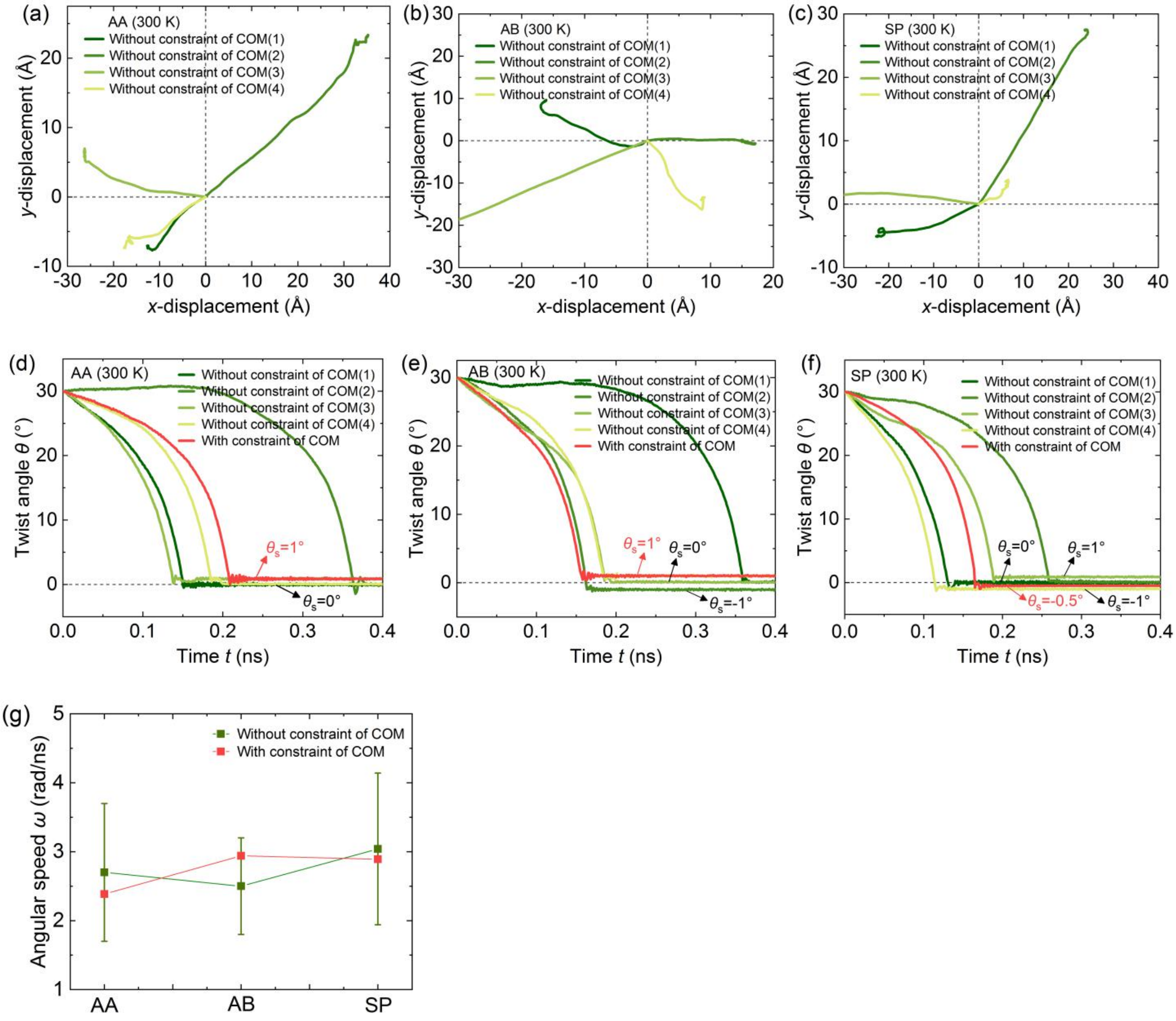

Figure S1. Flake rotation without/with the in-plane constraint of COM. (a-c) The flake experiences in-plane sliding. The initial position of the COM is at [0, 0]. (d-f) Twist angles θ as a function of the time t. (g) Comparison of the angular speed with/without the in-plane constraint of COM.

2. Heterostrain modulated twisting of graphene flake without constraint of COM

Taking the AA stacking model as an example, we applied biaxial loading/unloading (10%) on the relaxed *h*-BN layer. The in-plane COM of the graphene flake is not constrained. As shown in **Figure S2a**, heterostrain realizes the dynamic twisting of the flake. The modulated angular range (-2.7°~1°) is slightly small than that in Figure 3a (-3°~2.5°) due to in-plane sliding of the flake that dissipates small amount of energy, see **Figure S2b**.

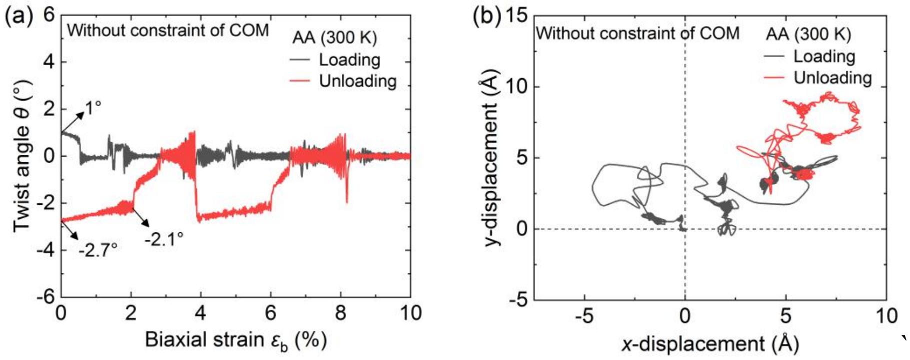

Figure S2. Heterostrain modulated dynamic twisting of the graphene flake without the in-plane constraint of COM. (a) The in-plane twisting, and (b) the in-plane sliding.
